# Gain- and Loss-of-Function Mutations in the Breast Cancer Gene *GATA3* Result in Differential Drug Sensitivity

**DOI:** 10.1371/journal.pgen.1006279

**Published:** 2016-09-02

**Authors:** Barbara Mair, Tomasz Konopka, Claudia Kerzendorfer, Katia Sleiman, Sejla Salic, Violeta Serra, Markus K. Muellner, Vasiliki Theodorou, Sebastian M. B. Nijman

**Affiliations:** 1 CeMM Research Center for Molecular Medicine of the Austrian Academy of Sciences, Vienna, Austria; 2 Ludwig Institute for Cancer Research, Nuffield Department of Clinical Medicine, University of Oxford, Oxford, United Kingdom; 3 Target Discovery Institute, Nuffield Department of Clinical Medicine, University of Oxford, Oxford, United Kingdom; 4 Experimental Therapeutics Group, Vall d'Hebron Institute of Oncology, Barcelona, Spain; 5 Institute of Molecular Biology and Biotechnology, Foundation for Research and Technology—Hellas, Heraklion, Crete, Greece; Dana Farber Cancer Institute, UNITED STATES

## Abstract

Patterns of somatic mutations in cancer genes provide information about their functional role in tumourigenesis, and thus indicate their potential for therapeutic exploitation. Yet, the classical distinction between oncogene and tumour suppressor may not always apply. For instance, *TP53* has been simultaneously associated with tumour suppressing and promoting activities. Here, we uncover a similar phenomenon for *GATA3*, a frequently mutated, yet poorly understood, breast cancer gene. We identify two functional classes of frameshift mutations that are associated with distinct expression profiles in tumours, differential disease-free patient survival and gain- and loss-of-function activities in a cell line model. Furthermore, we find an estrogen receptor-independent synthetic lethal interaction between a GATA3 frameshift mutant with an extended C-terminus and the histone methyltransferases G9A and GLP, indicating perturbed epigenetic regulation. Our findings reveal important insights into mutant GATA3 function and breast cancer, provide the first potential therapeutic strategy and suggest that dual tumour suppressive and oncogenic activities are more widespread than previously appreciated.

## Author Summary

Cancer is a disease caused by genetic mutations. Mutation patterns are often indicative of a gene’s function as either tumour promoting or tumour suppressive. Here we describe the frequently mutated, but poorly studied, breast cancer gene *GATA3* as a rare exception: We discover that two different functional classes of mutations in this gene can lead to either gain- or loss-of-function activities. The most common type of mutations, resulting in an unusually extended protein, is associated with differential gene expression and decreased disease-free survival. This mutant, in contrast to other mutations or the non-mutated protein, renders cells specifically vulnerable to inhibitors of two chromatin-modifying enzymes, the histone methyltransferases G9A and GLP. Our findings shed light on the functional consequences of frequent *GATA3* mutations in breast cancer and represent a first lead toward personalised therapy for a large subgroup of breast cancer patients.

## Introduction

High-throughput genome sequencing has allowed the systematic analysis of the complex mutational landscape of tumours and has provided key insights into tumour evolution and cancer etiology [1–3]. Mutation patterns in individual genes also reveal important insights into their role in tumourigenesis and can assist in distinguishing driver from passenger mutations [1–4].

Mutation rates are elevated in protein domains or regulatory sites, indicating their functional importance for cancer development [5,6]. It is typically assumed that all mutations within an individual gene have the same downstream consequences for tumourigenesis. However, at least one notable example challenges this paradigm. Distinct mutations in the *TP53* gene (encoding p53) lead to both loss-of-function and gain-of-function, impinging on multiple different pathways [7–10]. Yet, it is unclear if this type of dual activity of mutant p53 represents an exceptional case or is more common. We hypothesised that mutations in different positions in a cancer gene may result in different downstream consequences. To investigate this, we developed an unbiased computational approach and applied it to breast cancer, as large publicly available data sets are available for this cancer type.

Breast cancer has been studied extensively in terms of its molecular and genetic markers. Its classification into subtypes according to expression of receptors and gene expression profiles is used for diagnostic and prognostic purposes and forms the basis for treatment decisions [11–17]. Breast cancer is genetically heterogeneous and only four driver genes are mutated in more than 10% of patients [18–25]: *PIK3CA* (encoding the catalytic subunit of PI3K), *CDH1* (encoding E-cadherin), *TP53*, and *GATA3* (encoding GATA-binding protein 3). While the roles of the pro-survival PI3K pathway, cell adhesion, and p53 as the guardian of the genome in tumourigenesis are well studied, comparatively little is known about the role of the equally commonly mutated gene *GATA3*. To some extent this is due to the relatively recent discovery of the high prevalence of *GATA3* mutations [19–22,26]. In addition, model systems (e.g., cell lines, animal models) to study GATA3 in breast cancer are lacking, hampering functional studies.

GATA3 is a member of the GATA family of transcription factors and forms homodimers that bind conserved hexanucleotide sequences containing the central GATA motif [27–29]. It is a master regulator of helper T cell specification [30] and plays a critical role in development and differentiation of various tissues, including the mammary gland [31–33]. During normal mammary development, GATA3, together with the estrogen receptor (ER) [34–37], controls differentiation of the luminal epithelium in the terminal end buds in the breast. In adult tissues, GATA3 helps to maintain the luminal identity [38–41].

The contribution of GATA3 to cancer is, in contrast, poorly understood. Most of our current knowledge regarding GATA3’s potential function in breast cancer has been revealed from genomic studies highlighting an ER/FOXA1/GATA3 co-operating network of transcription factors in luminal tumours [14] and ER-positive cell line models [34,35,37,42,43]. Yet, the observation of *GATA3* downregulation during tumour progression and predominant frameshift mutations have led to the view that *GATA3* acts primarily as a tumour suppressor [44,45].

In this study, we identify differential functional consequences of mutation types in *GATA3*. We present evidence that the most common mutation type results in a protein with elongated C-terminus that displays effects consistent with gain-of-function activity in a cell line model. This is highly surprising, as frameshift mutations are generally believed to yield inactive proteins due to premature termination of translation. In addition, we describe a synthetic lethal interaction between this GATA3 mutant and drugs targeting the histone methyltransferases G9A and GLP, providing a first putative therapeutic opportunity for patients carrying *GATA3* mutations. Together, our findings demonstrate that different mutations in the same gene can result in differential drug sensitivities and contest the view that *GATA3* acts only as a tumour suppressor.

## Results

### Mutation positions in breast cancer genes are associated with differentially expressed genes

**Fig 1:**
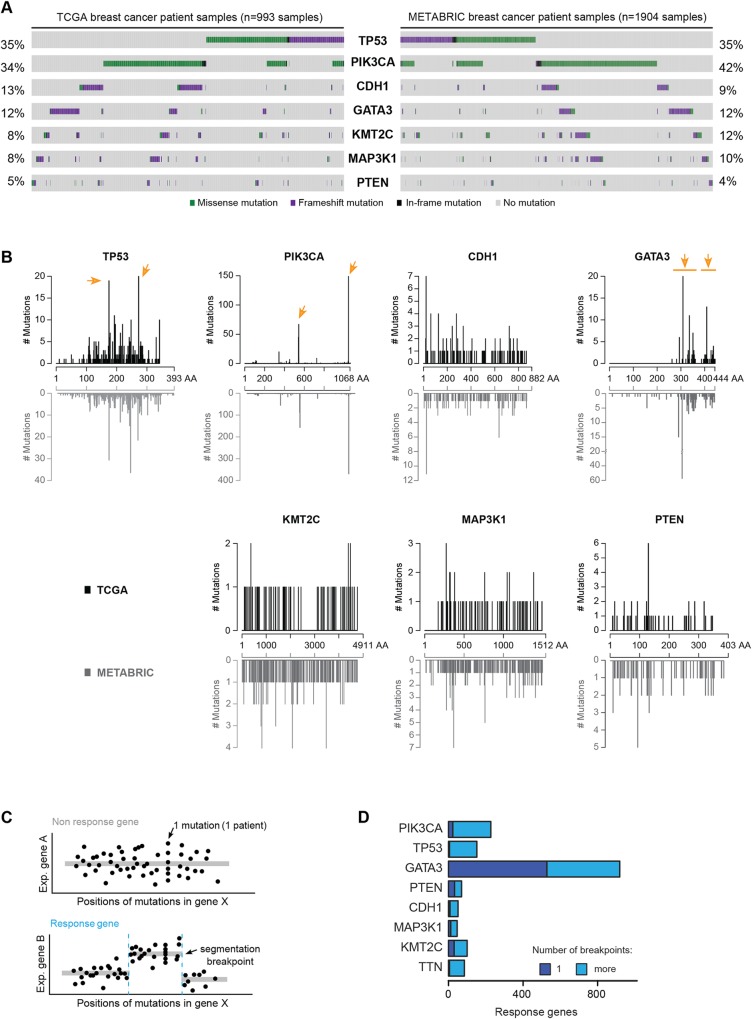
Breast cancer mutations are associated with domain-specific gene expression changes. **(A)** The most frequently mutated breast cancer genes from the TCGA (left) and METABRIC (right) cohorts are displayed. Bars from left to right indicate individual samples coloured by mutation type. Gray indicates absence of mutation. **(B)** Distribution of mutations along the indicated proteins. On top (black), TCGA data; at bottom (gray), METABRIC data. Hotspots (as determined by visual inspection and literature curation) are indicated with arrows. **(C)** Sketch of computational detection of mutational gene segmentation. TCGA expression and mutation data feed a segmentation analysis for a gene X. On top, expression of gene A is unrelated to the position of mutations. At bottom, a relation between mutation position along gene X and expression of gene B is detected. **(D)** Overview of expression changes in response to mutated domains in eight commonly mutated genes (TCGA data). Response genes are genes showing a segmentation pattern. *TTN* was included as a control gene.

To study mutation patterns in breast cancer, we used publicly available data from The Cancer Genome Atlas (TCGA) [23] and from the Molecular Taxonomy of Breast Cancer International Consortium (METABRIC) [25]. Fig 1A shows the most commonly mutated genes in breast cancer. Somatic mutations in these recurrently mutated breast cancer genes are often mutually exclusive [46,47] (Fig 1A, S1 Table) and distributed in a non-uniform fashion along the gene body (Fig 1B). The observed patterns are largely consistent between the TCGA and METABRIC datasets. For instance, *PIK3CA* mutations chiefly occur at just two positions corresponding to different protein domains: E545 in a helical regulatory domain and H1047 in the kinase domain [48]. Clear hotspot mutations at single amino acid residues or within narrow regions are also present in *TP53*, and to some extent in *GATA3* [49]. Mutations in *GATA3* (Entrez Gene ID: 2625) have not yet been extensively characterised, but the non-uniform distribution and mutual exclusivity with mutations in other cancer genes are strong indicators that *GATA3* is a cancer driver gene [25,50] (Fig 1B, S1 Table).

In order to assess potential functional consequences of regional mutation patterns, we devised an unbiased, systematic approach for linking the position of a mutation within a cancer gene with gene expression data. We reasoned that such an analysis could highlight domains in cancer genes that–when mutated–would result in differential downstream effects. First, we extracted from TCGA the genomic position for each mutation found in a patient in the seven selected driver genes, and the non-driver control gene *TTN* [51], along with the gene expression profiles from the same patients. Next, we used a segmentation approach to identify regions within a driver gene that led to a change in expression levels of another gene (see Materials and Methods). The identification of such patterns would suggest that the mutations in a particular region of the gene are functionally distinct. We termed genes that displayed altered expression along distinguishable segments of driver mutations “response genes” and refer to the border between two segments as a “segmentation breakpoint” (Fig 1C). Strikingly, we found that the highest number of response genes was associated with *GATA3* mutations (Fig 1D, S2 Table), where more than 900 genes displayed a segmented pattern. In comparison, around 200 response genes were linked with *PIK3CA* mutations, and fewer than 100 response genes were identified for the non-driver control gene *TTN*. The observation that the majority of response genes displayed a single breakpoint (Fig 1D) suggests that patient-derived *GATA3* mutations can be divided into two functionally distinct regions and that mutations in these regions are associated with differential gene expression in tumours.

### Different GATA3 frameshift mutation types are functionally distinct and affect disease-free survival

**Fig 2:**
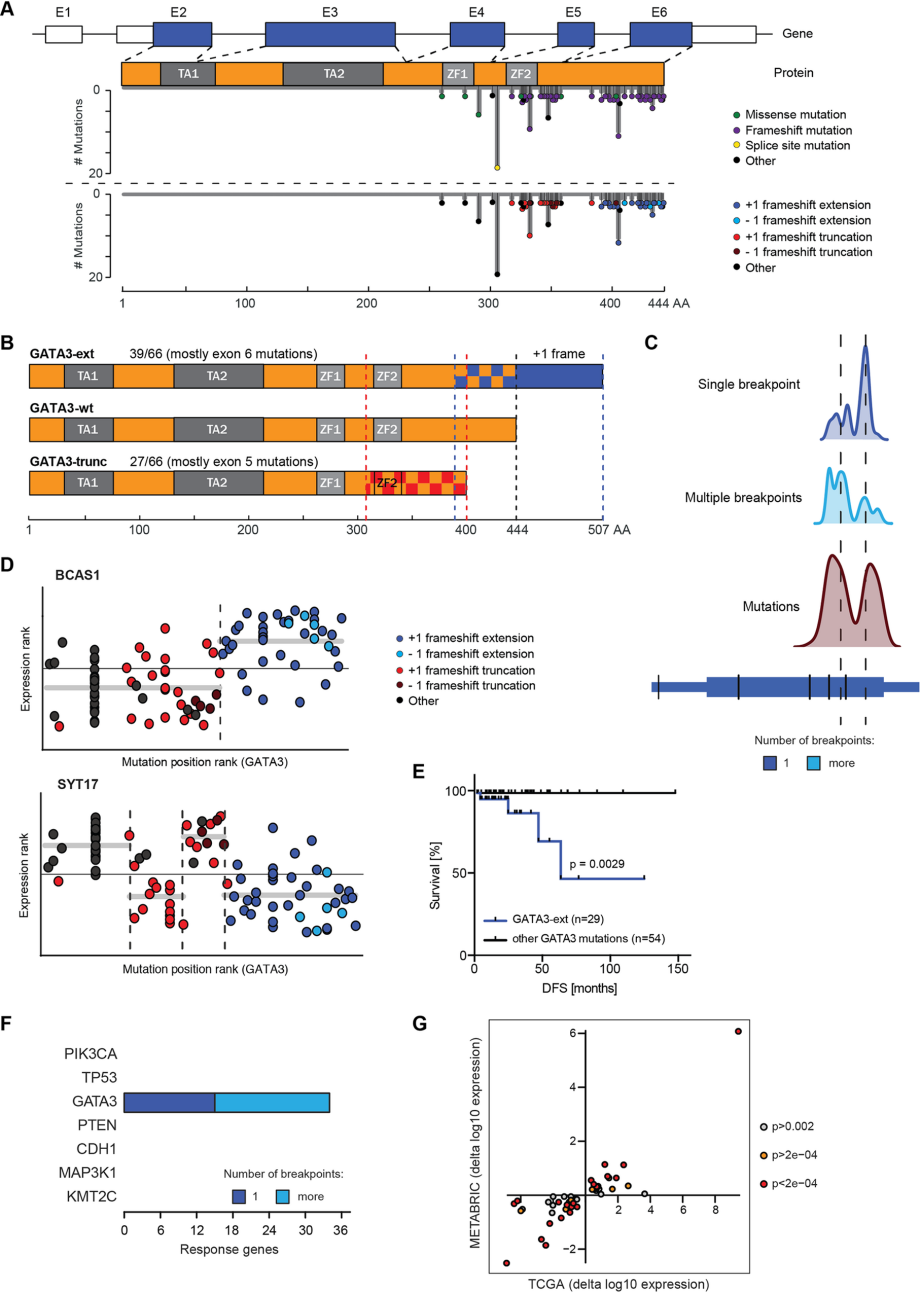
*GATA3* mutations are non-randomly distributed and fall into functionally distinct subclasses. **(A)** Schematic of *GATA3* gene and protein. TCGA mutations are coloured by class and/or type of frameshift and are mapped onto gene and protein length. Indicated are exons (E1-E6) and protein domains (TA, transactivation; ZF, zinc finger). **(B)** Sketch of GATA3 proteins. Wild-type and the two main protein products of frameshift mutants are depicted. Frameshift mutations affect the proteins in checkered areas. The predominant C-terminal extension is displayed in blue. Frequencies of mutations in the TCGA cohort are indicated. **(C)** Domain analysis for *GATA3*. Density plots show the smoothened distributions of segmentation breakpoints (single breakpoint, top; multiple breakpoints, middle) and mutations (bottom) in relation to genomic location. **(D)** Association between mutation position and expression of *BCAS1* and *SYT17* in the TCGA cohort. Horizontal axis shows ranked position of mutations along the *GATA3* gene. On the vertical axis, ranked normalised expression values are displayed. These values are then segmented as described. Mutations are coloured according to category as in (A). **(E)** Disease-free-survival (DFS) analysis of TCGA patients with GATA3-ext mutations vs. all other *GATA3* mutation classes. P-value was calculated with Log-rank (Mantel-Cox) test. **(F)** Overview of response genes showing a segmentation pattern in METABRIC data. Analysis was performed on the 46-gene TCGA patient-derived GATA3-ext signature. **(G)** Fold changes of GATA3-ext vs. other GATA3 mutant tumours in TCGA and METABRIC for 46 signature genes. Each dot represents one gene, colouring is by p-value (Wilcoxon) in METABRIC analysis.

Most *GATA3* mutations (66/99; 67%) in the TCGA dataset are heterozygous frameshift mutations in exon 5 and exon 6 (Fig 2A). Frameshifts in general lead to premature stop codons, which can substantially disrupt protein function. Indeed, approximately 41% (27/66) of the frameshift mutations in *GATA3* are predicted to result in an early stop codon (Fig 2B). These truncated proteins (hereafter referred to as GATA3-trunc) are stable and expressed in tumours [52], and, as GATA3 likely forms a homodimer [29], it is probable that they may act in a dominant negative manner [53,54]. These mutations would thus be consistent with a haplo-insufficient tumour suppressor function of GATA3.

Interestingly, most (39/66, 59%) frameshift mutations in *GATA3* result in a protein with an extended C-terminus. These extension mutations occur predominantly (33/39, 85%) in exon 6 and affect the resulting mutant proteins starting from different residues between alanine 395 and glycine 444, with a hotspot (11/39) at proline 409 (Fig 2A and 2B) [49]. The mutations are strongly biased toward the +1 frame (Fig 2A, bottom). This is surprising, as -1 frameshifts in this position would result in a shortened and aberrant C-terminus. The alternative +1 frame alters up to 49 amino acids of the original C-terminus and extends the protein by 63 novel amino acids (hereafter GATA3-ext, Fig 2B). Because frameshift mutations in the TCGA dataset as a whole do not display a frame preference, the bias toward the +1 frame in *GATA3* is suggestive of positive selection. One potential explanation for this could be that the GATA3-ext mutation is functionally distinct from other (truncating) mutations, for instance by providing a gain-of-function. Together, this demonstrates that our analysis can identify functional distributions of mutations as well as novel candidate tumourigenic mechanisms.

We next revisited our segmentation analysis to investigate the positional distributions of mutations and segmentation breakpoints (S1A–S1H Fig). In *GATA3*, the distribution of breakpoints present in single-breakpoint response genes was distinct from the distribution of all mutations (Fig 2C). It sharply peaked at a position that separated GATA3-ext from GATA3-trunc mutations. This is illustrated with *BCAS1*, the strongest *GATA3* response gene (Fig 2D, S3 Table). This indicates that genes like *BCAS1* are differentially expressed in tumours that contain the GATA3-ext mutation but not in tumours harbouring the GATA3-trunc mutation. Other response genes such as *SYT17* displayed more complex patterns, but one of the breakpoints often tended to separate the extension and truncation mutants (Fig 2D). Thus, the differential expression of response genes can functionally define the type of mutation. Together with the observation that the +1 frameshift mutations are under positive selection, this suggests that these GATA3-ext mutations are mechanistically distinct.

To investigate what functional aspects of cancer cells specifically correlate with GATA3-ext mutations, we calculated the association of this mutation class with gene expression levels without performing segmentation. We divided patients carrying *GATA3* mutations into two groups: those with GATA3-ext mutations and those with all other types. Corroborating the segmentation analysis, we obtained differentially expressed genes between these groups (S4 Table), which matched many of the previously identified response genes. This confirms that cellular processes are indeed differentially affected in GATA3-ext tumours in comparison to the other *GATA3* mutant tumours.

Differential gene expression in GATA3-ext tumours may indicate distinct tumour characteristics and this could affect disease progression and therapeutic response. To address this, we used the previously defined patient groups and performed survival analysis of the TCGA patients. Only GATA3-ext patients progressed during the follow-up period of the cohort but no patients with an alternative *GATA3* mutation did. Accordingly, patients with GATA3-ext mutations displayed significantly (p = 0.0029) shortened disease-free survival in the TCGA cohort (Fig 2E). This indicates that GATA3-ext is a putative biomarker for disease progression and is consistent with the notion that extension mutants have important mechanistic properties that are distinct from other *GATA3* mutations.

To assess whether GATA3-ext mutations are associated with similar expression changes in an independent patient cohort, we analysed the METABRIC dataset. The METABRIC cohort carries relatively fewer GATA3-ext mutations and displays a moderately different mutation distribution in *GATA3* (compare lower panels of S1D with S1I Fig). Interestingly, substantial differences in *GATA3* mutation patterns have also been noted in a Chinese cohort [46] (see Discussion), suggesting that genetic background and environmental factors play an important role in GATA3-driven breast cancer. We further noted that in the METABRIC cohort disease-free survival was similar for patients harbouring GATA3-ext or other GATA mutations, further indicating considerable differences in cohort composition (S1J Fig).

Despite these dissimilarities, we repeated the segmentation analysis in both the METABRIC and TCGA datasets using the GATA3-ext gene signature derived from TCGA (S4 Table). The analysis was limited to 46/50 genes, as expression data for four genes were not present in the METABRIC cohort. As expected, all 46 genes showed segmentation for GATA3 in the TCGA dataset (S1K Fig). Notably, 34/46 signature genes qualified as GATA3-specific response genes in the independent METABRIC dataset (Fig 2F, S1I Fig). This implies that these genes are differentially expressed in GATA3-ext tumours. Next, we calculated the fold change of the 46 genes in GATA3-ext samples relative to all other *GATA3* mutant tumours. Strikingly, the changes in both datasets occurred in consistent directions for all 46 genes (Fig 2G), indicating qualitative agreement between TCGA and METABRIC for the GATA3-ext gene signature. Hence, the distinctive effects of GATA3-ext are recapitulated in an independent breast cancer patient cohort.

### GATA3 mutant expression in cell lines

Following investigations of *GATA3* mutation types in human patient data, we wished to study these mutations types *in vitro* in order to understand their functional implications in more detail. An endogenous heterozygous truncating mutation in exon 5 (cDNA:1006insG) in the commonly used MCF7 breast cancer cell line has been previously reported and was shown to decrease DNA binding and increase protein half-life [53,55]. However, we did not find cancer cell lines with GATA3-ext mutations by mining the Cancer Cell Line Encyclopaedia (CCLE) [56] and the Catalogue of Somatic Mutations in Cancer (COSMIC) [57] databases or by analysing 45 breast cancer cell lines by Sanger sequencing and Western blot. The high frequency of *GATA3* mutations in breast tumours is thus not well represented in cell lines. We also tested tumour tissue from 10 luminal patient-derived xenograft (PDX) mouse models and did not detect any GATA3-ext mutations either. Although the small sample numbers preclude a strong conclusion, together these results suggest that GATA3-ext mutant cells may not adapt well to *ex vivo* culture conditions.

The lack of cell lines with naturally occurring GATA3-ext mutations impelled us to search for an alternative model system. To distinguish putative gain-of-function from dominant negative effects, we wished to study GATA3-ext in the absence of wild-type GATA3. We attempted to inactivate the endogenous locus by CRISPR/Cas9 gene editing in several ER-positive breast cancer cell lines (MCF7, T47D and CAMA1), but this did not yield viable homozygous null clones (~150 clones analysed). CRISPR/Cas9-directed replacement of endogenous *GATA3* by GATA3-ext was equally unsuccessful (>100 clones analysed). This suggests that at least one copy of wild-type *GATA3* is required for viability in these cell lines, which is in accordance with the findings from human cancer samples but complicates the introduction of a mutated version for *in vitro* models.

**Fig 3:**
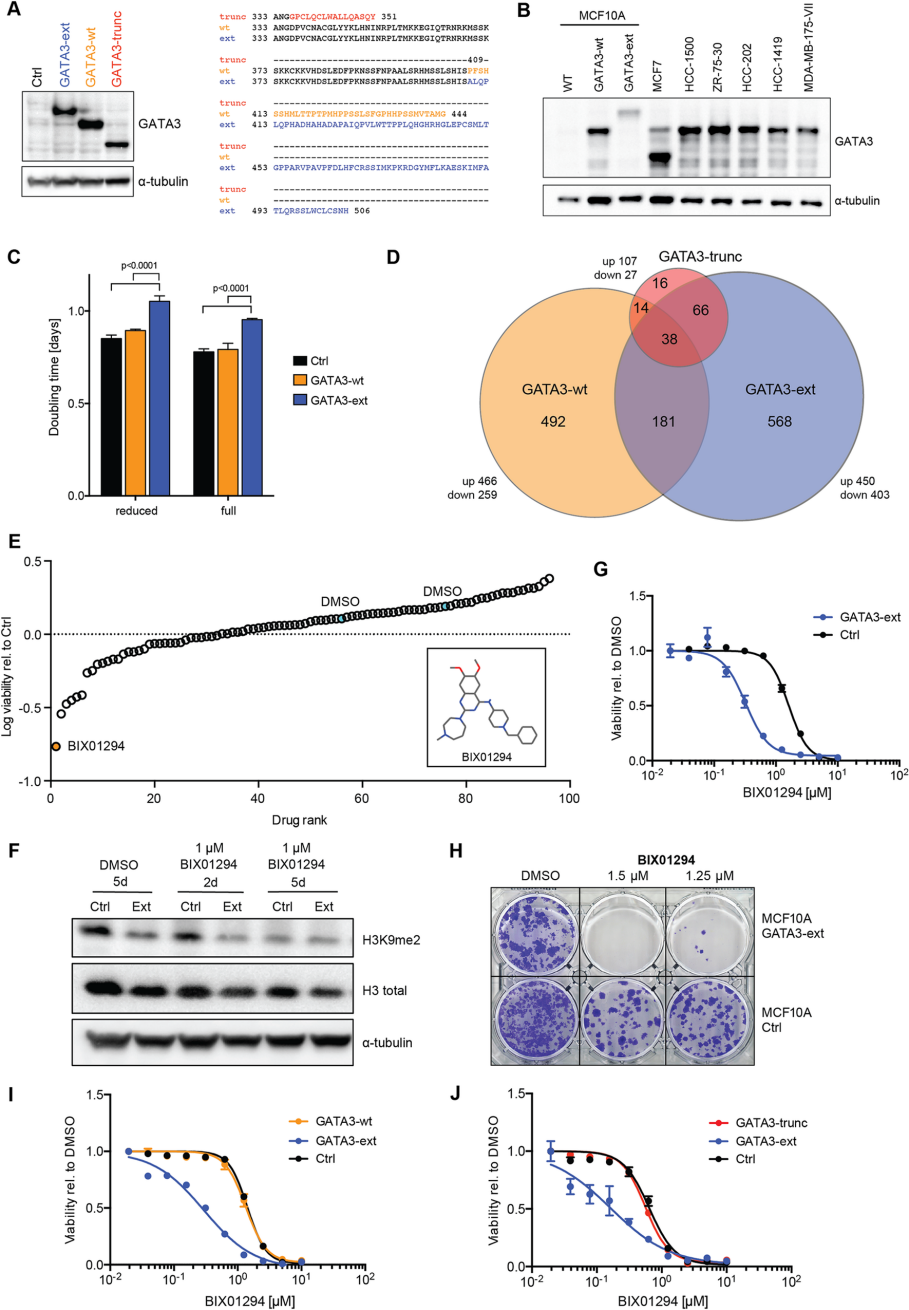
A GATA3 extension mutant cell line model is sensitive to G9A/GLP inhibition. **(A)** Western blot shows expression of wild-type and mutant GATA3 proteins in MCF10A cells. Protein sequences of canonical and alternative C-termini are displayed. **(B)** Western blot shows endogenous GATA3 levels in various breast cancer cell lines in comparison to MCF10A wild-type and GATA3-ext and -wt expressing cells. **(C)** Doubling time of MCF10A control, GATA3-ext and -wt cells as measured over 12 days. Cells were cultured in full and reduced supplement conditions (see Materials and Methods), counted every 4 days and cumulative cell numbers and doubling times were calculated. P-values were calculated with 2-way ANOVA with Holm-Sidak’s multiple comparisons correction. **(D)** Differentially expressed genes and their overlap from RNA sequencing of MCF10A GATA3-wt, GATA3-ext and GATA3-trunc cells (all compared to control cells). **(E)** Small-molecule screen in MCF10A control and GATA3-ext cells. Cells were treated with drugs for 6 days before viability was measured. Data were median-centred and normalised to control. Top hit and negative controls (DMSO) are indicated. Inset shows the structure of BIX01294. **(F)** Western blot shows decrease of histone 3 lysine 9 dimethylation (H3K9me2) upon treatment of MCF10A control and GATA3-ext cells with BIX01294 for 2 or 5 days. **(G-J)** Dose response curves (DRC) and colony formation assay (CFA) in reduced supplement-containing medium. MCF10A control and cells expressing GATA3-ext, GATA3-wt or GATA3-trunc were treated with the indicated concentrations of BIX01294 for 4 days (DRC) or 10 days (CFA). Cell viability was measured by luminescent ATP read-out and normalised to a DMSO control (DRC) or cells were fixed and stained with crystal violet (CFA). The graphs show the mean of triplicate measurements. Error bars indicate SEM.

To establish an alternative model, we used the non-tumourigenic MCF10A breast epithelial cell line that naturally expresses very low protein levels of endogenous GATA3 (Fig 3A and 3B). We stably expressed wild-type GATA3 (GATA3-wt), GATA3-ext (cDNA:1224insG; p:P409fs) and GATA3-trunc (cDNA:1006insG; p:G336fs) through retroviral transduction and puromycin selection (Fig 3A). The GATA3-ext protein was stable, albeit expressed at slightly lower levels than GATA3-wt. Importantly, the expression levels of the GATA3 proteins were in the physiological range of endogenous GATA3 observed in various other breast cancer cell lines (Fig 3B). Furthermore, confocal microscopy showed nuclear localisation for both mutants as well as for the wild-type protein (S2A Fig).

We noted a slight but significant decrease in growth rate of MCF10A GATA3-ext cells as compared to GATA3-wt (Fig 3C). This was consistent between independent infections of the parental MCF10A cells with titrated virus, excluding an effect of the viral transduction itself. The specific effect of GATA3-ext shows that this mutation affects MCF10A cells’ ability to proliferate in standard tissue culture medium conditions.

We next performed RNA sequencing on MCF10A GATA3-wt, GATA3-ext, GATA3-trunc or control vector expressing cells to characterise the effects of GATA3 mutations at the cellular level. The expression of GATA3-wt and GATA3-ext resulted in up- or downregulation of 725 and 853 genes, respectively, with respect to the control (p <0.05, FC >1.5), indicating widespread transcriptional changes. In contrast, expression of GATA3-trunc yielded a considerably smaller signature (134 genes), which could be indicative of loss-of-function (Fig 3D, S5 Table). The majority of the GATA3-wt and GATA3-ext signatures consisted of uniquely regulated, non-overlapping genes (Fig 3D, S2B Fig). Accordingly, gene ontology (GO) analysis revealed significant enrichment of gene sets relating to unique terms for each of the GATA3 constructs (S5 Table). For instance, the GATA3-wt gene set is enriched for cytokine-linked processes, whereas the GATA3-ext signature shows a significant enrichment for peptidyl-tyrosine modification processes. These results indicate that expression of GATA3-ext and GATA3-trunc invoke starkly distinct changes in gene expression, and the large number of uniquely regulated genes in GATA3-ext cells supports a gain-of-function of this mutant.

We found a small 4-gene overlap between the TCGA and MCF10A GATA3-ext signatures (S2B and S2C Fig). We validated one of these, the triglyceride metabolism gene *PNPLA3*, in an independent set of experiments by qRT-PCR and observed consistent downregulation in cells expressing GATA3-ext. (S2D and S2E Fig). This is in agreement with patient tumour data (S2F Fig). Yet, the observation that most signature genes derived from the ER-negative MCF10A cell line model do not overlap with the patient data reflects the well-known biological differences between patient tumour samples and cell culture model systems.

Together, these data indicate that GATA3-ext is functionally active upon overexpression and that GATA3-ext and GATA3-trunc mutants are mechanistically different from each other and from the wild-type protein.

### GATA3-ext cells are sensitive to G9A/GLP inhibition

Chemical genetic interactions can reveal therapeutic vulnerabilities and pinpoint cellular processes that are affected by mutant proteins [58,59]. Therefore, we performed a chemical genetic screen to identify compounds that specifically affect GATA3-ext cells. We assembled a small-molecule library containing ~100 approved and experimental anti-cancer drugs, and a number of tool compounds. We used MCF10A cells expressing the GATA3-ext protein or a control vector and exposed them to compounds for 6 days before measuring viability. To mimic limited nutrient supply in a tumour and to render the cells more responsive to drugs, cells were treated under reduced media supplement conditions (S3A and S3B Fig, S6 Table).

The compound that most strongly reduced GATA3-ext viability was BIX101294 [60,61], a specific inhibitor of the histone 3 lysine 9 methyltransferases G9A (*EHMT2*) and GLP (G9A-like protein; *EHMT1*) (Fig 3E, S6 Table). As expected, BIX01294 reduced global histone 3 lysine 9 dimethylation (H3K9me2) in MCF10A-GATA3-ext and control cells over time (Fig 3F).

We validated this unexpected interaction with short- and long-term treatment and in both full and reduced media supplement conditions (Fig 3G and 3H and S3C Fig). The effective concentration resulting in a 50% inhibition of viability (EC_50_) for cells expressing the GATA3-ext mutant was consistently 5- to 10-fold lower than in control cells.

To determine if this sensitivity was specific for GATA3-ext, we tested the compound on MCF10A cells expressing GATA3-wt or GATA3-trunc. The sensitivity of these cells to the compound was identical to control cells infected with an empty vector (Fig 3I and 3J). Accordingly, MCF7 cells, which heterozygously express GATA3-trunc, display average sensitivity to BIX101294 when compared to a panel of 25 other breast (cancer) cell lines (S3D Fig). Next, we wished to rule out that the observed effects of GATA3-ext overexpression were due to a dominant negative effect. To address this, we depleted endogenous GATA3 by shRNAs and tested if this could phenocopy GATA3-ext expression. The knockdown did not result in enhanced sensitivity to BIX101294 (S4A Fig). We thus conclude that the sensitivity arises from a specific interaction between the drug and the extended GATA3 protein.

GATA3-ext mutations in patients are predominantly heterozygous, and as endogenous GATA3 protein levels in MCF10A cells are very low, we co-expressed GATA3-ext and GATA3-wt to assess whether the presence of GATA3-wt alters the differential toxicity of BIX0124. GATA3-ext+wt cells were equally sensitive as GATA3-ext cells (S4B Fig). This experiment suggests that the GATA3-ext-induced BIX01294 sensitivity is independent of the presence of a wild-type *GATA3* allele.

Together, these data further highlight functional differences between GATA3 truncation and extension mutants and imply that extension mutants act by a mechanism that is different from typical loss-of-function or dominant negative effects.

### GATA3-ext cell sensitivity is due to on-target inhibition of G9A/GLP

G9A and GLP are histone methyltransferases (HMTs) that form a heterodimer and catalyse specific mono- and di-methylation at histone 3 lysine 9 (H3K9) [62]. Di-methylation of this residue is associated with transcriptional repression and has been demonstrated to occur aberrantly at tumour suppressor genes, often coinciding with upregulation of *G9A* [63]. In the TCGA dataset, *EHMT1* and *EHMT2* are not differentially expressed in GATA3-ext tumours and do not show a segmentation pattern (S5 Fig).

**Fig 4:**
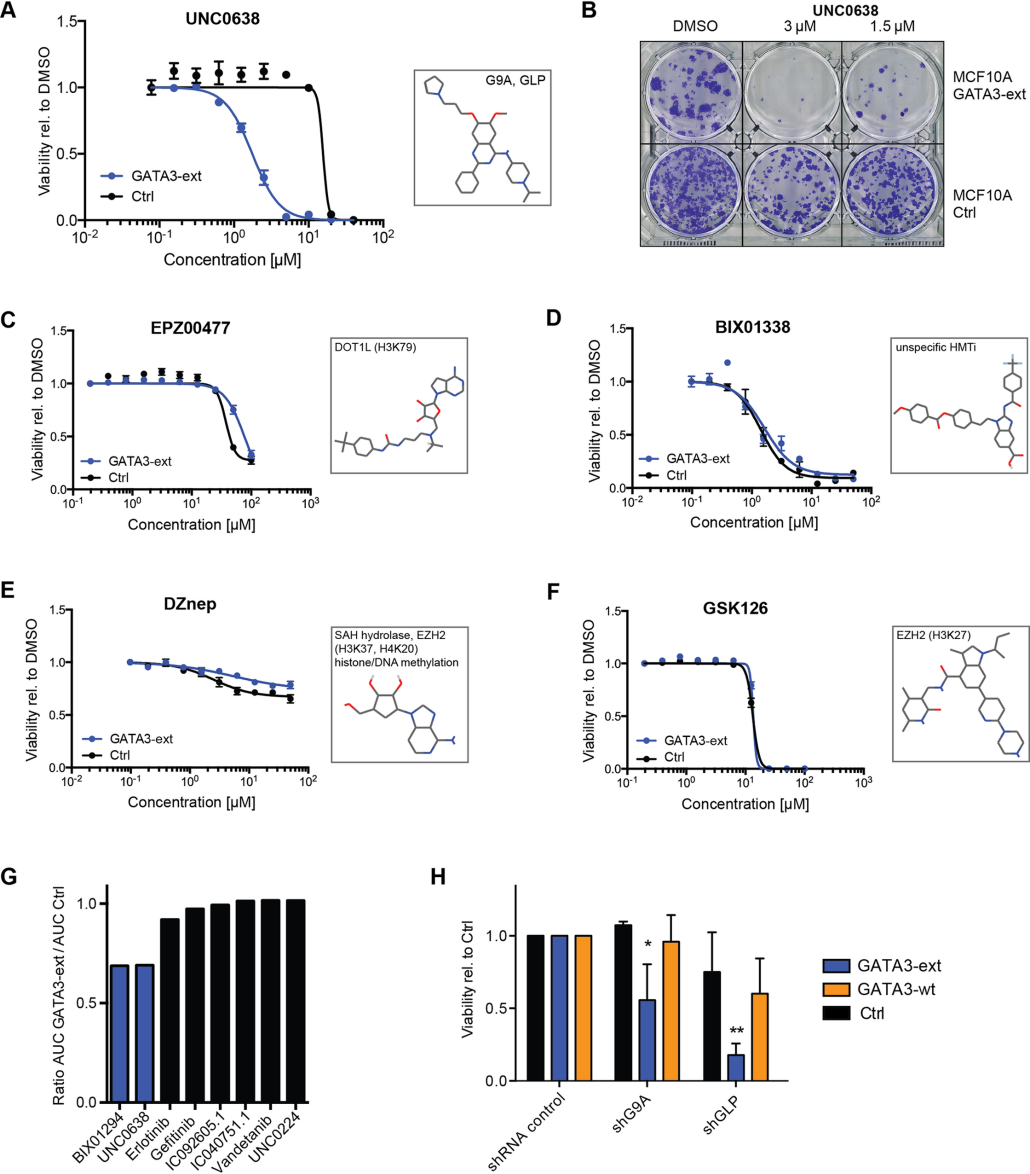
GATA3-ext cell sensitivity is due to on-target inhibition of G9A/GLP. **(A-G)** Dose response curves (DRC) and colony formation assay (CFA) in reduced supplement-containing medium. MCF10A control and cells expressing GATA3-ext were treated with the indicated concentrations of HMT inhibitors (A-F) or structurally related quinazoline compounds (G) for 4 days (DRC) or 10 days (CFA). Cell viability was measured and normalised to a DMSO control (DRC) or cells were fixed and stained with crystal violet (CFA). The graphs show the mean of triplicate measurements. Error bars indicate SEM. Boxes show compound structures and intended targets (corresponding histone modifications in brackets). AUC, Area under the curve. **(H)** MCF10A control, GATA3-ext or GATA3-wt cells were infected with lentivirus encoding shRNAs for G9A and GLP and subsequently selected with hygromycin for 4 days. Cell viability was measured and normalised to the control. The graphs show the mean of three independent experiments. Error bars indicate SD. P-values were calculated with 2-way ANOVA with Holm-Sidak's multiple comparisons correction. *p<0.05, **p<0.005.

To assess the specificity of the synthetic interaction between GATA3-ext and G9A/GLP, we tested a second G9A/GLP inhibitor (UNC0638 [64]). Although this compound did not score as a hit in the screen, possibly due to a suboptimal screening concentration, repeated validation showed a similar degree of hypersensitivity of GATA3-ext cells (Fig 4A and 4B and S3C Fig, S4 Fig). Next, we tested a set of inhibitors of various other HMTs and did not detect differential sensitivity (Fig 4C–4F), suggesting that the interaction with GATA3-ext does not occur with histone methyltransferase activity in general. Further, GATA3-ext and control cells were equally responsive to other structurally similar quinazoline compounds not targeting G9A/GLP, consistent with a specific and on-target effect of BIX01294 and UNC0638 (Fig 4G, S6A Fig). In order to verify the involvement of G9A and GLP more directly, we depleted them by shRNA in GATA3-wt, GATA3-ext and control cells. Only the viability of GATA3-ext cells was significantly affected (Fig 4H, S6B Fig), suggesting that both enzymes contribute to the sensitivity to BIX01294 and UNC0638.

### G9A/GLP inhibitor sensitivity is due to increased apoptosis and is independent of estrogen receptor signalling

**Fig 5:**
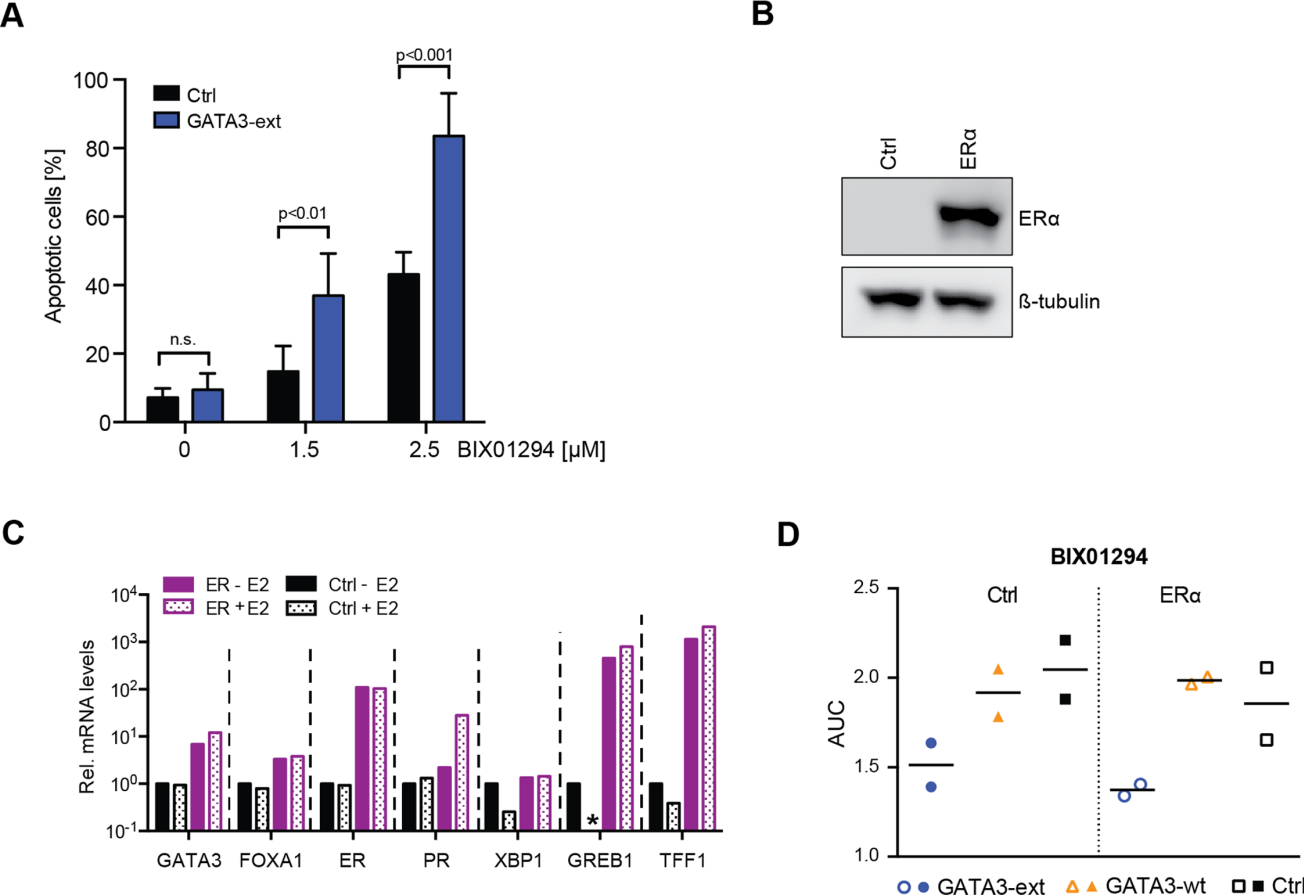
G9A/GLP inhibitor sensitivity is due to increased apoptosis and independent of ER signalling. **(A)** Apoptotic (Annexin V positive) MCF10A control or GATA3-ext cells were measured by FACS after treatment with BIX01294 for 5 days. Error bars indicate SD; n = 3 independent experiments. P-values were calculated with two-tailed unpaired t-tests with Holm-Sidak's multiple comparisons correction; n.s., not significant. **(B)** Western blot shows expression of ERα after transduction into MCF10A cells. **(C)** qRT-PCR analysis of ER target genes in control and ER-transduced MCF10A cell lines with/without supplementation of 10nM β-estradiol (E2) for 6 hours after starvation. mRNA levels were normalised to *GAPDH* and displayed relative to control cells without E2. * = 0.074. **(D)** MCF10A control and ER-transduced cell lines expressing GATA3-ext or control were treated with 0–10μM BIX01294 for 4d. Cell viability was measured and normalised to a DMSO control. Area under curve (AUC) was calculated for two independent experiments of triplicate measurements.

To characterise the mechanisms underlying the sensitivity of GATA3-ext cells to G9A/GLP inhibition, we first analysed potential cell cycle effects upon BIX01294 treatment. We did not observe a difference in cell cycle progression between GATA3-ext and control cells as assessed by BrdU incorporation or DNA content (S7 Fig). However, GATA3-ext cells were more prone to undergo apoptosis upon drug treatment than control cells (Fig 5A).

As GATA3 is functionally linked with ER expression and activity [34–37], we also assessed the impact of ER signalling on sensitivity to G9A/GLP inhibition in GATA3-ext cells. We expressed ERα in our MCF10A model and confirmed that ER target genes were induced upon ER expression and/or treatment with the ER agonist β-estradiol (E2) (Fig 5B and 5C). The sensitivity of GATA3-ext cells to G9A/GLP inhibition was not significantly influenced by the level of ER signalling (Fig 5D), suggesting a mechanism that is independent from previously described functional interactions of GATA3.

## Discussion

Recent studies have begun to address the role of GATA3 in breast cancer. GATA3 has been suggested as a negative regulator of epithelial-to-mesenchymal transition and metastasis but putative tumour promoting effects have also been reported [26,45,53–55,65–76]. Critically, these studies have almost exclusively focussed on wild-type GATA3 and only a few have studied GATA3 truncating mutations. To our knowledge, our study is the first that highlights and addresses the most frequent GATA3 mutation type, i.e. mutations resulting in an extended C-terminal protein.

Protein-extending mutations in cancer genes are unusual but not unprecedented. Recently, frameshift extension mutations in *CALR* (encoding calreticulin) were identified in myeloproliferative neoplasms [77] and *WT1* extension mutants have been described in Wilms kidney tumours [78].

Cancer driver mutations are often divided into gain-of-function and loss-of-function mutations. Loss-of-function mutations result in an inactive or less active protein, whereas gain-of-function mutations lead to a more active protein or acquisition of a different function. Several observations in our study indicate that GATA3-ext proteins are mechanistically distinct from other GATA3 mutants and GATA3 wild-type, hinting toward a gain-of-function:

First, GATA3-trunc mutants lack a larger part of the normal GATA3 protein sequence than GATA3-ext. This makes it rather unlikely that GATA3-ext is more perturbed in its normal physiological function than other GATA3 mutants.

Second, in patients, GATA3-ext is associated with the differential expression of a distinct group of response genes that is not affected by other GATA3 mutants. Differential effects on gene expression were also observed in the MCF10A cell line model expressing GATA3-ext and GATA3-trunc.

Third, we have found differences in outcome for patients harbouring GATA3-ext mutations, at least in the TCGA cohort. There, GATA3-ext is associated with reduced disease-free survival compared to other *GATA3* mutations, suggesting that these tumours display a different pathology with respect to recurrence. Of note, all *GATA3* mutations together correlated with improved disease-free and overall survival in a Chinese patient cohort [46]. *GATA3* mutations as a whole displayed a marginally significant trend to improved overall survival only in ER-positive patients in the TCGA and METABRIC cohorts [25,46] but not in a smaller Dutch study [76]. Interestingly, *GATA3* frameshift mutations were strongly underrepresented in the Chinese cohort (22% vs. 78% missense mutations) as compared to TCGA (93% vs. 7%). The authors suggest different mutational evolution of luminal breast cancer in different populations as an explanation for these discrepancies, with few Asian patients being included in the TCGA cohort [46]. However, these studies do not discriminate between GATA3-ext, GATA3-trunc or other GATA3 mutations. Our survival analysis indicates that indeed this separation is important, as only GATA3-ext mutations are associated with reduced disease-free survival.

Fourth, we observe strong genetic selection for +1 frameshift mutations, leading to one specific C-terminal extension.

Fifth, GATA3-ext is stable in cells and displays functional characteristics (e.g., drug sensitivities) that are not observed in cells expressing other GATA3 proteins or cells in which GATA3 is depleted.

Taken together, these lines of evidence provide substantial support for the hypothesis that GATA3-ext adopts certain neomorphic functions that might replace or act in addition to its wild-type properties. Importantly, our findings challenge the view that GATA3 only acts as a tumour suppressor that is downregulated or inactivated in breast cancer [14–16,19–26,79].

This GATA3-ext gain-of-function hypothesis parallels *TP53* mutations in certain aspects, including gain- and loss-of-function in the same gene. For this reason, we have adopted the gain-of-function terminology in analogy to p53 and propose to label GATA3-truncation mutations as primarily loss-of-function and GATA3-extension mutations as gain-of-function. Like GATA3, p53 is a transcription factor that acts as a homo-oligomer, and hence, gain-of-function mutations do not necessarily imply a constitutively active form of the protein, as it is observed for many kinase gain-of-function mutants. Instead, a plethora of different functions for oncogenic p53 have been described, including altered subcellular localization, changed DNA-binding affinities and a different spectrum of binding partners and target genes. Ultimately, these activities can lead to enhanced proliferation, inhibition of apoptosis, chemoresistance, or increased invasiveness [8–10].

It remains unclear how GATA3-ext exerts its specific activity. It has been postulated [80] that the GATA3 C-terminus is essential for maintaining protein stability but we did not observe strong differences upon ectopic expression in MCF10A cells. Therefore, an alternative mechanism is likely to underpin GATA3-ext function. For instance, GATA3-ext may display differential binding partners or altered DNA binding sites.

The GATA3-ext protein rendered cells sensitive to inhibition of the G9A and GLP histone methyltransferases. G9A and GLP are upregulated in a number of cancers, correlating with higher H3K9me2 levels and silencing of tumour suppressor genes [63]. Intriguingly, wild-type GATA3 and G9A have been recently found to physically interact [65]. The biochemical and functional interaction of GATA3 with histone methyltransferases may explain the changes of active histone modifications and altered enhancer accessibility in breast cancer cells depleted of GATA3 [37]. Yet, if and how this relates to drug sensitivity specifically in GATA3-ext expressing cells remains unclear.

Our MCF10A cell line model does not fully recapitulate the context of *GATA3* mutations in tumours in several ways, among them the heterozygous mutation state and the ER status. Due to lack of a more appropriate model system, we addressed these concerns separately by co-expression and knock-down experiments of mutant and wild-type *GATA3* and *ESR1* (encoding ER). Even though RNA sequencing data from the MCF10A model only show a marginal overlap with the TCGA patient-derived GATA3-ext signature on an individual gene level, we believe that the MCF10A cell line model provides a valid context to study basic mechanistic differences between GATA3-wt, GATA3-ext and GATA3-trunc. The patient data and MCF10A model agree in that GATA3-ext and GATA3-trunc mutants act in a mechanistically different manner from each other and from the wild-type protein. We believe that this finding is biologically and potentially clinically relevant despite the exact mechanisms not yet being understood. In this regard, the identified synthetic lethal interaction between GATA3-ext and G9A/GLP inhibition provides the first clinically testable hypothesis for application of these drugs and the first lead for a treatment of this major subgroup of breast cancer patients. Thus, further pre-clinical study of the uncovered gene-drug interaction is warranted.

Together, our study provides important insights into the function and potential druggability of one of the most frequent breast cancer mutants and a striking example of how different mutations in the same cancer driver can result in distinct downstream consequences.

## Materials and Methods

### Plasmids, shRNAs and cloning

The *GATA3-ext* cDNA sequence was synthesised by Epoch Life Science and shuttled into Gateway-compatible pBABE-puro or -neo vectors. *GATA3-wt* and *GATA3-trunc* were generated using the QuikChange II Site-Directed Mutagenesis kit in the same plasmid. The *ESR1* (ER) sequence was obtained from pEGFP-C1-ER (gift from Michael Mancini, Addgene plasmid #28230) and Gateway-cloned into pBABE-neo. Most shRNA sequences were obtained from The RNAi Consortium (TRC); shGAT3_1 was in pSicoR; shGATA3_2 and shGATA3_3 were cloned into pLKO.1-puro; shG9A_1, shG9A_2, shGLP_1, and shGLP_2 were cloned into pLKO.1-hygro, which was derived from pLKO.1-puro by replacing the puromycin with a hygromycin cassette (BamHI/NsiI). If not indicated otherwise, corresponding empty vectors were used as controls. The control cDNA for the small-molecule screen (pBABE-puro TBX3) was a gift from Thijn Brummelkamp. shRNA sequences are provided in S6 Table.

### Cell lines

MCF10A cells were purchased from ATCC and grown in DMEM/F12 medium (Gibco) +5% horse serum (Gibco) +0.02μg/ml EGF (Sigma) +0.5μg/ml hydrocortisone (Sigma) +0.1μg/ml cholera toxin (Sigma) +10μg/ml insulin (Gibco) +1% penicillin/streptomycin (Gibco) (= full supplements). In “reduced supplement conditions”, all ingredients except antibiotics were used at 20% of their original concentration. MCF7 cells were obtained from ATCC and grown in DMEM medium +10% FCS (Gibco or Sigma) +1% penicillin/streptomycin (Gibco). All other cell lines (except HMEC, which were a gift from Christoph Gebeshuber) were obtained from ATCC and cultured in recommended conditions (S6 Table). Cells were cultured at 37°C and 5% CO_2_ (except for cells in Leibovitz’s L-15 medium, which were cultured without CO_2_) and regularly tested for mycoplasma infection.

Stable cell lines were generated by retro- or lentiviral infection and subsequent selection with puromycin (2μg/ml, Sigma) or neomycin (geneticin, 500μg/ml, Gibco) for at least 3 days. Viral particles were produced by transfecting the retro- or lentiviral vector and corresponding packaging plasmids (encoding polymerase and envelope proteins) into HEK293-T cells. The supernatant was harvested 48–72 hours post transfection and its virus titer was determined on MCF10A cells. Target cells were then infected with an approximate MOI of 1 in presence of 7–10μg/ml polybrene (Sigma, Millipore).

### Viability assays and growth curves

MCF10A cells were seeded in reduced supplement conditions (unless indicated otherwise) and treated with drugs or DMSO for 4 days (dose response curves) or 10 days (colony formation assays) in triplicates. All other cell lines were seeded in their respective full media and treated for 3–11 days until reaching 90% confluency. Alternatively, cells were infected with lentivirus (shGLP/shG9A) on the next day and subsequently selected with hygromycin (50μg/ml, Sigma) for 4 days. Cell viability was measured by luminescent ATP read-out (CellTiterGlo, Promega) and normalised to the control (plotted as lowest concentration point to allow log-calculation), or cells were fixed using 3.7% paraformaldehyde and stained with 0.1% crystal violet in 5% ethanol. Data analysis and Area under curve (AUC) calculations were performed in GraphPad Prism.

For measuring doubling time, cells were seeded at defined numbers (CASY Cell Counter, OMNI Life Science; BioRad TC20 Automated Cell Counter), counted every 4 days and re-seeded. Cumulative cell numbers and doubling times were calculated in Microsoft Excel and GraphPad Prism.

### Compounds and small-molecule screen

The majority of small-molecules used in this study were purchased from SYNthesis med chem, Selleck and Sigma. Erastin, imatinib and erlotinib were gifts from Georg Winter and Giulio Superti-Furga, TH588 from Ulrika Warpman Berglund and Thomas Helleday, and DBZ JQ-1, IC092605.1 and IC040751.1 from James Bradner. Further information on vendors/sources and concentrations is provided in S6 Table.

For the small-molecule screen, cells were seeded in 384-well plates in reduced supplement conditions and compounds were added the next day in quadruplicates at concentrations equalling a previously determined EC_20_. After 6 days, viability was measured as described.

Drawings of chemical structures were generated using Avogadro [81].

### Apoptosis analysis

Cells were treated with BIX01294 or DMSO for 5 days and then stained with propidium iodide (0.02μg/μl; Sigma) and AnnexinV AlexaFlour 647 (BioLegend) in Binding Buffer (BioLegend) according to manufacturer’s instructions. 20,000–30,000 cells were analysed by FACS (BD FACSCalibur). Data analysis was performed with BD CellQuest Pro, FlowJo and GraphPad Prism. Camptothecin (3μM for 16 hours; Sigma) was used as a positive control.

### Quantitative real-time PCR (qRT-PCR)

Total RNA was isolated using the QIAGEN RNeasy Mini kit, DNase digested (Ambion TURBO DNA-free), normalised by concentration and 0.2–1μg were reverse transcribed with random hexamer primers (Fermentas RevertAid Reverse Transcriptase kit). qRT-PCR was performed in triplicates with 1μl cDNA using KAPA SYBR FAST Master Mix on an AppliedBiosystems StepOne Plus or 7900HT Fast Real-Time PCR System. mRNA levels were normalised to *GAPDH* and displayed relative to the control cell line (ΔΔCt method). Primer sequences are provided in S6 Table. For the analysis of ER target genes, cells were starved for 16 hours and treated with 10nM β-estradiol (E2) for 6 hours in absence of serum and growth factors before total RNA was isolated.

### Western blotting

Cells were counted and lysed in reducing sample buffer by boiling. Proteins were separated by SDS-PAGE on 4–12% Bis-Tris-Gels (Invitrogen) and then transferred to Amersham Hybond PVDF membranes (GE Healthcare). Blocking and antibody incubations were carried out in 0.2% I-Block (Tropix) in PBST and membranes were washed with PBS +0.1% Tween-20. HRP-coupled secondary antibodies (goat anti-rabbit or anti-mouse, BioRad, 1:10,000) were detected with Western Lightning ECL Plus (Perkin Elmer) and visualised using a BioRad ChemiDoc or MF-ChemiBIS 3.2 (DNR Bio-Imaging Systems) imaging system. Antibody details are provided in S6 Table.

### RNA sequencing

Total RNA was isolated from MCF10A cells (2 independent infections per condition) using the QIAGEN RNeasy Mini kit and DNase digested (Ambion TURBO DNA-free). 1μg was used as input for library preparation with the Illumina TruSeq RNA Sample Kit v2 according to manufacturer’s instructions. cDNA concentrations were measured using Qubit dsDNA HS assay on a Qubit 2.0 Fluorometric Quantitation System (Life Technologies). 15ng of amplified libraries were pooled, analysed for size distribution using an Agilent Tapestation 2200 D1000 and quantified using Picogreen. Sequencing was performed on a single lane as 75bp paired end reads on a HiSeq4000 according to Illumina specifications. Approximately 30 million reads were generated per sample. The sequences were demultiplexed, quality controlled with FastQC v0.10.1 and aligned to hg19 using STAR v2.4.2a [82]. Gene counts were obtained with featureCounts (Subread, v1.4.5-p1) [83]. Differential gene expression analysis was performed in R using the packages edgeR v3.12.1 [84], GeneOverlap v1.6.0 (Shen and Sinai, 2013) and topGO v2.22.0 (org.Hs.eg.db v3.2.3 and GO.db v3.2.2) (Alexa and Rahnenfuhrer, 2016). Venn diagrams were produced using BioVenn [85].

All raw sequencing data have been deposited in the European Nucleotide Archive under project PRJEB14813.

### Cell cycle analysis

Cells were treated with 1μM BIX01294 for 3 days, followed by a 15-minute BrdU pulse. As control, an S phase arrest was induced with 1μM Camptothecin (Sigma) for 4 hours prior to BrdU incorporation. Cells were fixed in ice-cold 70% ethanol for 24 hours and resuspended in PBS containing 0.5% Tween-20, 10μg/ml propidium iodide and 500μg/ml RNaseA. Incorporated BrdU was detected using a FITC-conjugated anti-BrdU antibody (Becton-Dickson) according to manufacturer’s instructions. 20,000 cells were analysed by FACS (BD FACSCalibur). Data analysis was performed with BD CellQuest Pro, FlowJo and GraphPad Prism.

### Immunofluorescence microscopy

For immunofluorescence microscopy, cells were plated onto coverslips (VWR) in a 24-well plate. Next day, cells were washed twice with ice-cold PBS and fixed with 4% PFA +0.1% Triton X-100 in PBS for 20 minutes on ice. Cells were permeabilised with 0.5% Triton X-100 in PBS for 20 minutes and blocked with 10% FCS +0.1% Triton X-100 in PBS for 1 hour with three washes between individual steps. Primary (GATA3 D13C9, Cell Signaling #5852, 1:100) and secondary (AlexaFluor 546 goat anti-rabbit, Invitrogen, 1:500) antibodies were diluted in blocking solution and incubated for 1 hour at RT. Finally, cells were stained with DAPI (10μg/ml, Sigma) for 10 minutes at RT in the dark. Slides were mounted in 85% glycerol and images were acquired on a Zeiss LSM 710 confocal imaging system.

### TCGA and METABRIC cohort analysis

Data for Figs 1A, 1B, 2A, 2B and 2E and S1J and S1 Table were downloaded and/or visualised through the cBioPortal for Cancer Genomics [86,87] (http://www.cbioportal.org; datasets “Breast Invasive Carcinoma, TCGA, Cell 2015, 1105 samples” and “METABRIC Breast Cancer, 1980 samples”). Survival analysis was performed in GraphPad Prism and R.

For the initial segmentation analysis based on TCGA, expression and mutation data of the BRCA cohort were downloaded from the TCGA data portal (https://tcga-data.nci.nih.gov/tcga/). Mutation data were subset to eliminate all mutations marked as “Silent”. For each candidate gene with high mutation load, the data were further trimmed to eliminate patients with more than one mutation in that gene. The mutation positions in the gene of interest were ranked and then compared to ranked, normalised expression levels of every expressed gene in the transcriptome. Segmentation was carried out with the R package DNAcopy [88] using a strict cut-off for definition of breakpoints (alpha = 0.005), a corresponding large number of permutations (nperm = 20000), and a strict minimum for segment length (min.width = 5). Genes whose expression across the cohort showed at least one breakpoint along the target gene were termed response genes. Breakpoints found through the segmentation analysis were visualised along the body of the gene using density plots. Densities were computed using breakpoint positions for genes showing one breakpoint and for those showing two breakpoints or more. For *GATA3*, details for mutation start site and mutation sequence were jointly used to classify patients into groups: protein truncations in the +1 and -1 frames, protein extensions in the +1 and -1 frame, and all other mutations; patients with multiple mutations were excluded. Gene expression comparisons were then performed between the dominant group (protein extensions in the +1 frame) and all other mutant groups combined. In each comparison, we evaluated fold changes by comparing median expression in each group and statistical significance through Wilcoxon tests.

For the validation analysis using the METABRIC dataset, mutation profiles on driver genes and expression data for the GATA3-ext signature genes were obtained from the METABRIC consortium (see Acknowledgments). Mutation profiles were subset to remove mutations marked as “Silent” as before; mutations marked as “RNA” (noncoding substitution variants in untranslated regions of the genes) were also removed. All other calculations were carried out using the same procedures as for the TCGA dataset.

To assess consistency between TCGA and METABRIC on the GATA3-ext signature, we compared fold changes between GATA3-ext and other GATA3-mutant patients in the two datasets. The comparison was targeted on the small gene signature obtained from the TCGA analysis.

## Supporting Information

S1 Fig Summary of Domain Analysis for Genes Commonly Mutated in Breast Cancer. **(A-H)** On top, density plots show the (smoothed) distributions of segmentation breakpoints in relation to each gene's exon structure. Densities are drawn separately for segmentations revealing one or more breakpoints. At bottom, density plots show the (smoothed) distributions of mutations in relation to the genomic location. TCGA data. **(I)** As (A-H) for *GATA3* in METABRIC data. **(J)** Disease-free-survival (DFS) analysis of METABRIC patients with GATA3-ext mutations vs. all other *GATA3* mutation classes. **(K)** Overview of response genes showing a segmentation pattern in TCGA data. Analysis was performed on the 46-gene GATA3-ext signature.
(TIF)

S2 Fig GATA3-ext in a Cell Line Model. **(A)** Immunofluorescence images (63-fold magnification, 1.5-fold zoom) show localisation of GATA3-wt, GATA3-ext and GATA3-trunc stably transduced into MCF10A cells. **(B)** Comparison of differentially expressed genes in MCF10A GATA3-wt, GATA3-ext and GATA3-trunc cells as compared to control. “Signature” indicates genes from TCGA patient-derived GATA3-ext signature, “random” means 800 (median all genes up + median all genes down) randomly selected genes from all genes expressed in MCF10A cells. Upper triangle displays p-values as calculated with Fisher’s exact test with Bonferroni correction (n.s., not significant), lower triangle displays number of overlapping genes. Colours scale with numerical values, numbers highlighted in red are used for (C). **(C)** Venn diagrams displaying the overlap of MCF10A GATA3-wt and GATA3-ext with TCGA patient-derived GATA3-ext signatures. Symbols for overlapping genes are indicated, *PNPLA3* is highlighted in red. No overlap was found with GATA3-trunc. **(D, E)** RNA sequencing (RPKM values, D) and qRT-PCR (E) analysis of *PNPLA3* mRNA levels in MCF10A GATA3-ext and GATA3-wt cells relative to control cells (set to 1). Data are aggregate from 2 (D) or 3 (E) independently transduced cell lines each. Error bars indicate SEM, p-value was calculated with a paired Student’s t-test. **(F)** Association between *GATA3* mutations and *PNPLA3* gene expression in patient data. Expression values are the normalised RSEM values provided by TCGA. P-values were calculated with Wilcoxon test.
(TIF)

S3 Fig Sensitivity of MCF10A and Other Breast (Cancer) Cell Lines to Drugs in General and to G9A/GLP Inhibition in Full vs. Reduced Media. **(A, B)** MCF10A cells were seeded in either full (100%) or reduced (20%) supplement-containing medium and treated with the indicated concentrations of different drugs (n = 60) for 4 days. Cell viability was measured and normalised to the mean of all DMSO controls on the same plate (n = 36). **(A)** The relative viability of each well is displayed colour-coded according to legend. **(B)** Box-plots summarising data in (A). Whiskers indicate minimum and maximum values, boxes represent 25th to 75th percentile, and the line indicates the median. P-values were calculated with Mann-Whitney U-test; n.s., not significant. **(C)** Dose response curves (DRC) in full (100%) and reduced (20%) supplement-containing medium. MCF10A control and cells expressing GATA3-ext or GATA3-wt were treated with the indicated concentrations of BIX01294 or UNC0638 for 4 days. Cell viability was measured and normalised to a DMSO control. The graphs show the mean of triplicate measurements. Error bars indicate SEM. **(D)** Breast (cancer) cell lines were treated with 0–20μM BIX01294 for 3-11d. Cell viability was measured and normalised to a DMSO control. Each dot represents the Area under curve (AUC) value for an independent experiment of triplicate measurements. Lines indicate median. Presence or absence of ER expression is indicated by filled or empty squares, respectively.
(TIF)

S4 Fig G9A/GLP Inhibitor Sensitivity upon Depletion or Co-Expression of GATA3. **(A)** Dose response curves (DRC) in reduced supplement-containing medium. MCF10A control cells and cells transduced with 3 different shRNAs targeting *GATA3* were treated with the indicated concentrations of BIX01294 or UNC0638 for 3–4 days. Cell viability was measured and normalised to a DMSO control. The graphs show the mean of triplicate measurements. Error bars indicate SEM. *GATA3* mRNA levels were analysed by qRT-PCR, normalised to *GAPDH* and displayed relative to control cells (right panel). Error bars indicate SD. **(B)** DRCs as in (A) using MCF10A cells expressing GATA3-ext with or without co-expression of GATA3-wt. Western blot (right panel) shows (co-)expression of wild-type and mutant GATA3 proteins in MCF10A cells.
(TIF)

S5 Fig *EHMT1* and *EHMT2* Levels Are Not Altered in GATA3-ext Tumours. **(A)** Association between *GATA3* mutations and *EHMT1/2* gene expression in patient data. Expression values are the normalised RSEM values provided by TCGA. n.s., not significant by Wilcoxon test. **(B)** Association between mutation position and expression of *EHMT1* and *EHMT2*. Horizontal axis shows ranked position of mutations along the *GATA3* gene. On the vertical axis, ranked normalised expression values are displayed. These values are then segmented as described. Mutations are coloured according to category.
(TIF)

S6 Fig MCF10A GATA3-ext Cell Sensitivity Is Specific to G9A/GLP Inhibition. **(A)** DRCs accompanying Fig 4G. MCF10A control and cells expressing GATA3-ext were treated with the indicated concentrations of structurally related quinazoline compounds for 4 days. Cell viability was measured and normalised to a DMSO control. The graphs show the mean of triplicate measurements. Error bars indicate SEM. Boxes show compound structures. **(B)** *G9A* and *GLP* mRNA levels in MCF10A cells transduced with shRNAs were analysed by qRT-PCR. Values were normalised to *GAPDH* and displayed relative to parental cells (i.e. Ctrl (cDNA) or GATA3-ext) transduced with shRNA control.
(TIF)

S7 Fig Cell Cycle Analysis in MCF10A GATA3-ext and Control Cells. **(A)** Cells were treated with DMSO, BIX01294 (1μM) for 3 days or Camptothecin (CPT, 1μM, positive control) for 4 hours. Bromodeoxyuridine (BrdU) was added, cells were fixed, stained with an antibody recognising BrdU and analysed by FACS. **(B)** Cells were fixed, stained with propidium iodide (PI) and analysed by FACS. Cell cycle phases were derived from DNA content.
(TIF)

S1 Table Mutual Exclusivity Analysis of Most Frequent Breast Cancer Mutations in TCGA and METABRIC Data. (XLSX)

S2 Table Response Genes from TCGA Segmentation Analysis of Eight Breast Cancer Genes. (XLSX)

S3 Table Top Response Genes from GATA3 TCGA Segmentation Analysis. (XLSX)

S4 Table Top Genes Associated with GATA3-ext in TCGA Patient Data. (XLSX)

S5 Table Differentially Expressed Genes and Top Associated GO-Terms in MCF10A GATA3-wt, GATA3-ext and GATA3-trunc Cells. (XLSX)

S6 Table Small-Molecule Screens and Detailed Information about Compounds, Primers, shRNAs, Cell Lines and Antibodies Used in This Study. (XLSX)
