# Supplementary figures and images for: Gain- and Loss-of-Function Mutations in the Breast Cancer Gene GATA3 Result in Differential Drug Sensitivity
Source: PLoS Genet. 2016 Sep 2;12(9):e1006279. doi: 10.1371/journal.pgen.1006279 (PMC5010247; doi:10.1371/journal.pgen.1006279)

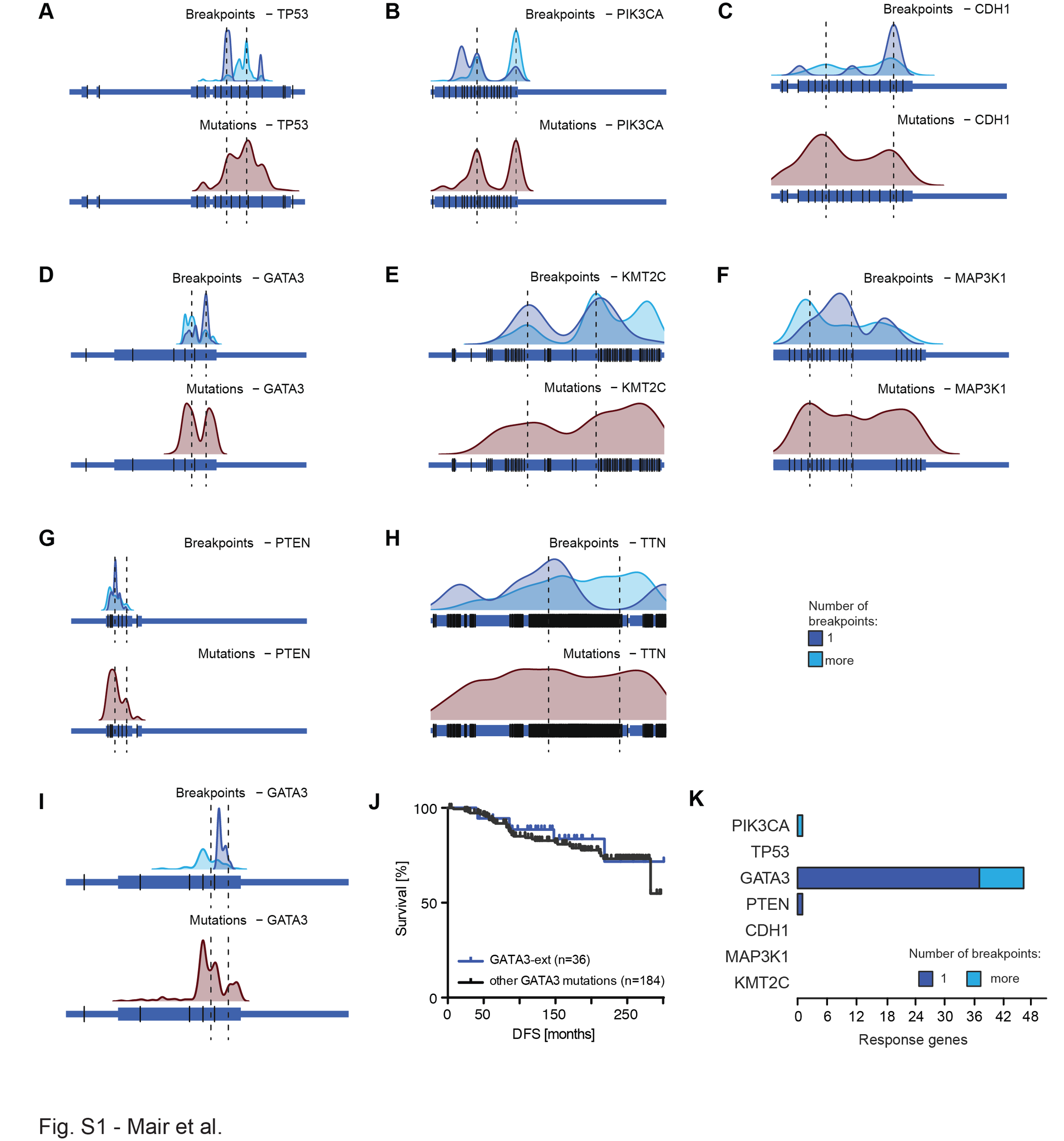

Supplement: S1 Fig — (A-H) On top, density plots show the (smoothed) distributions of segmentation breakpoints in relation to each gene's exon structure. Densities are drawn separately for segmentations revealing one or more breakpoints. At bottom, density plots show the (smoothed) distributions of mutations in relation to the genomic location. TCGA data. (I) As (A-H) for GATA3 in METABRIC data. (J) Disease-free-survival (DFS) analysis of METABRIC patients with GATA3-ext mutations vs. all other GATA3 mutation classes. (K) Overview of response genes showing a segmentation pattern in TCGA data. Analysis was performed on the 46-gene GATA3-ext signature. (TIF) [file pgen.1006279.s001.tif]

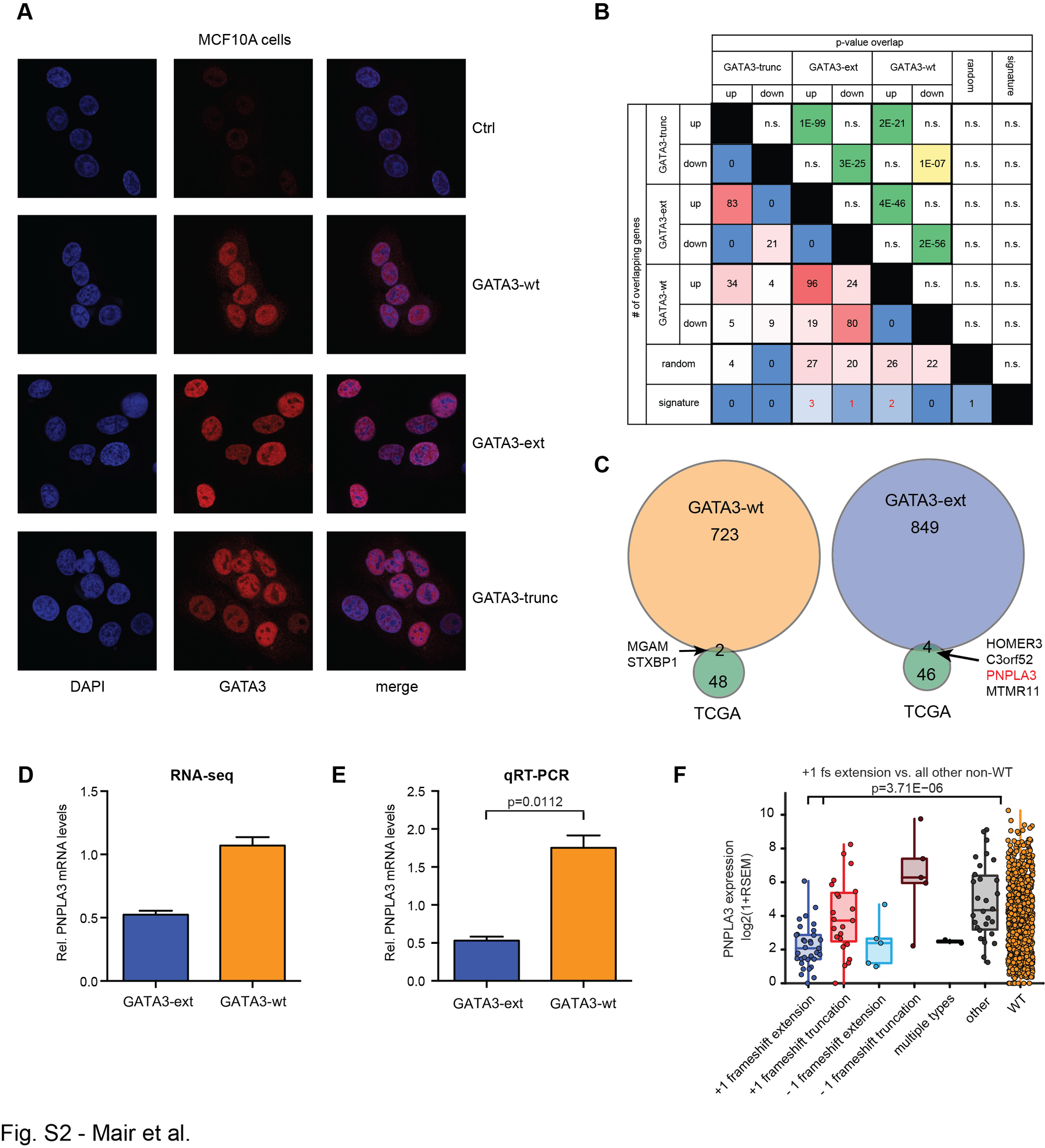

Supplement: S2 Fig — (A) Immunofluorescence images (63-fold magnification, 1.5-fold zoom) show localisation of GATA3-wt, GATA3-ext and GATA3-trunc stably transduced into MCF10A cells. (B) Comparison of differentially expressed genes in MCF10A GATA3-wt, GATA3-ext and GATA3-trunc cells as compared to control. “Signature” indicates genes from TCGA patient-derived GATA3-ext signature, “random” means 800 (median all genes up + median all genes down) randomly selected genes from all genes expressed in MCF10A cells. Upper triangle displays p-values as calculated with Fisher’s exact test with Bonferroni correction (n.s., not significant), lower triangle displays number of overlapping genes. Colours scale with numerical values, numbers highlighted in red are used for (C). (C) Venn diagrams displaying the overlap of MCF10A GATA3-wt and GATA3-ext with TCGA patient-derived GATA3-ext signatures. Symbols for overlapping genes are indicated, PNPLA3 is highlighted in red. No overlap was found with GATA3-trunc. (D, E) RNA sequencing (RPKM values, D) and qRT-PCR (E) analysis of PNPLA3 mRNA levels in MCF10A GATA3-ext and GATA3-wt cells relative to control cells (set to 1). Data are aggregate from 2 (D) or 3 (E) independently transduced cell lines each. Error bars indicate SEM, p-value was calculated with a paired Student’s t-test. (F) Association between GATA3 mutations and PNPLA3 gene expression in patient data. Expression values are the normalised RSEM values provided by TCGA. P-values were calculated with Wilcoxon test. (TIF) [file pgen.1006279.s002.tif]

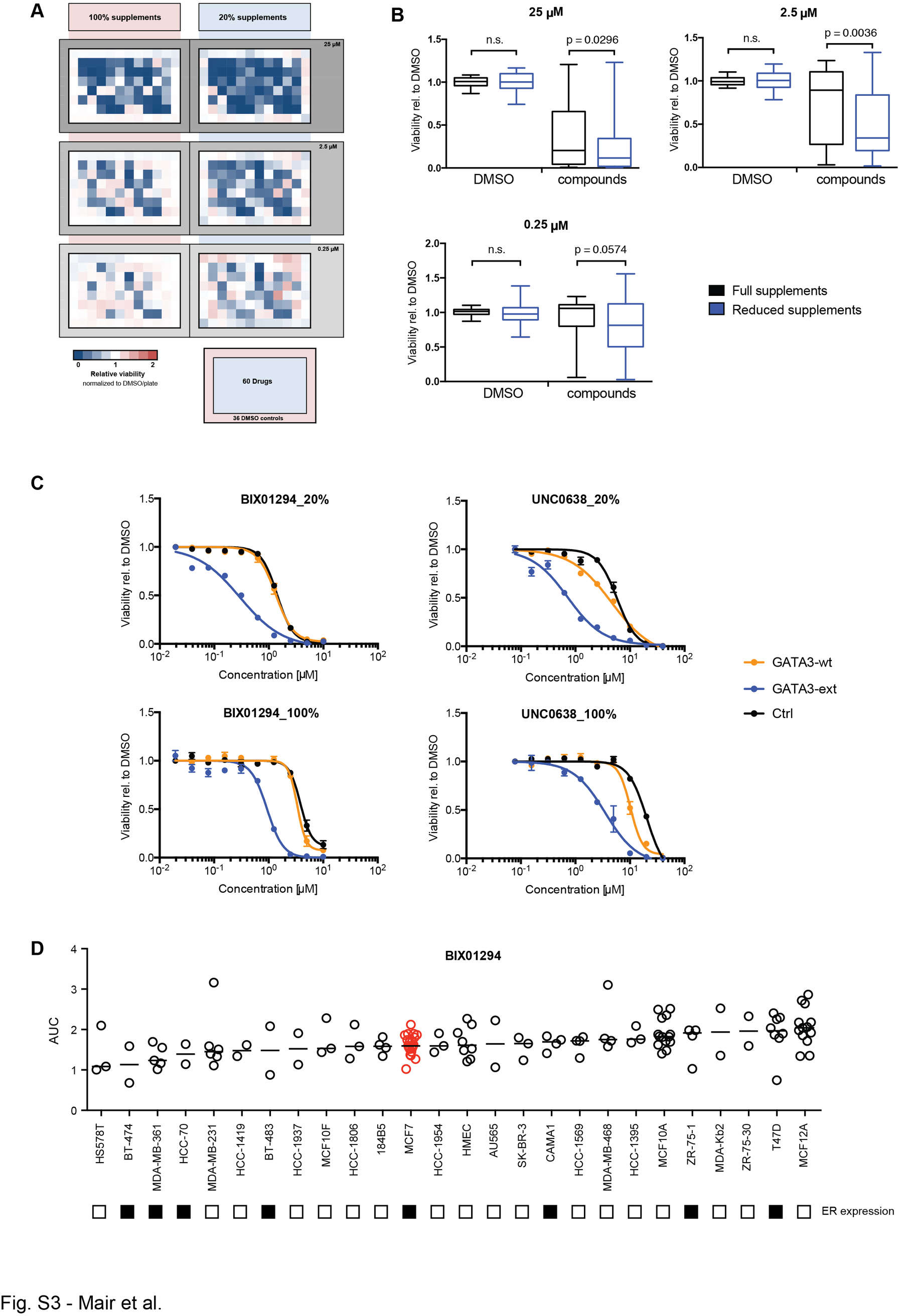

Supplement: S3 Fig — (A, B) MCF10A cells were seeded in either full (100%) or reduced (20%) supplement-containing medium and treated with the indicated concentrations of different drugs (n = 60) for 4 days. Cell viability was measured and normalised to the mean of all DMSO controls on the same plate (n = 36). (A) The relative viability of each well is displayed colour-coded according to legend. (B) Box-plots summarising data in (A). Whiskers indicate minimum and maximum values, boxes represent 25th to 75th percentile, and the line indicates the median. P-values were calculated with Mann-Whitney U-test; n.s., not significant. (C) Dose response curves (DRC) in full (100%) and reduced (20%) supplement-containing medium. MCF10A control and cells expressing GATA3-ext or GATA3-wt were treated with the indicated concentrations of BIX01294 or UNC0638 for 4 days. Cell viability was measured and normalised to a DMSO control. The graphs show the mean of triplicate measurements. Error bars indicate SEM. (D) Breast (cancer) cell lines were treated with 0–20μM BIX01294 for 3-11d. Cell viability was measured and normalised to a DMSO control. Each dot represents the Area under curve (AUC) value for an independent experiment of triplicate measurements. Lines indicate median. Presence or absence of ER expression is indicated by filled or empty squares, respectively. (TIF) [file pgen.1006279.s003.tif]

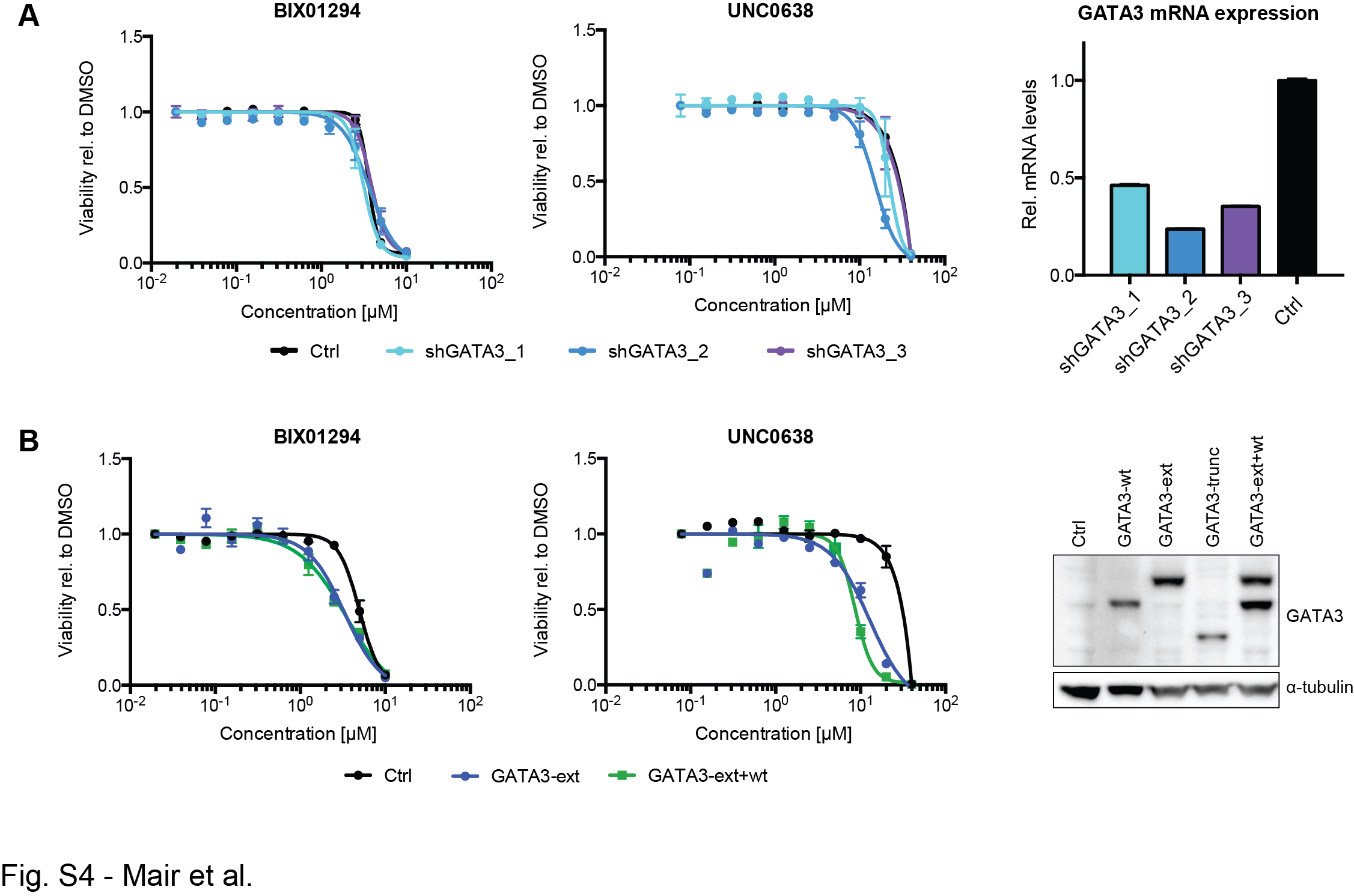

Supplement: S4 Fig — (A) Dose response curves (DRC) in reduced supplement-containing medium. MCF10A control cells and cells transduced with 3 different shRNAs targeting GATA3 were treated with the indicated concentrations of BIX01294 or UNC0638 for 3–4 days. Cell viability was measured and normalised to a DMSO control. The graphs show the mean of triplicate measurements. Error bars indicate SEM. GATA3 mRNA levels were analysed by qRT-PCR, normalised to GAPDH and displayed relative to control cells (right panel). Error bars indicate SD. (B) DRCs as in (A) using MCF10A cells expressing GATA3-ext with or without co-expression of GATA3-wt. Western blot (right panel) shows (co-)expression of wild-type and mutant GATA3 proteins in MCF10A cells. (TIF) [file pgen.1006279.s004.tif]

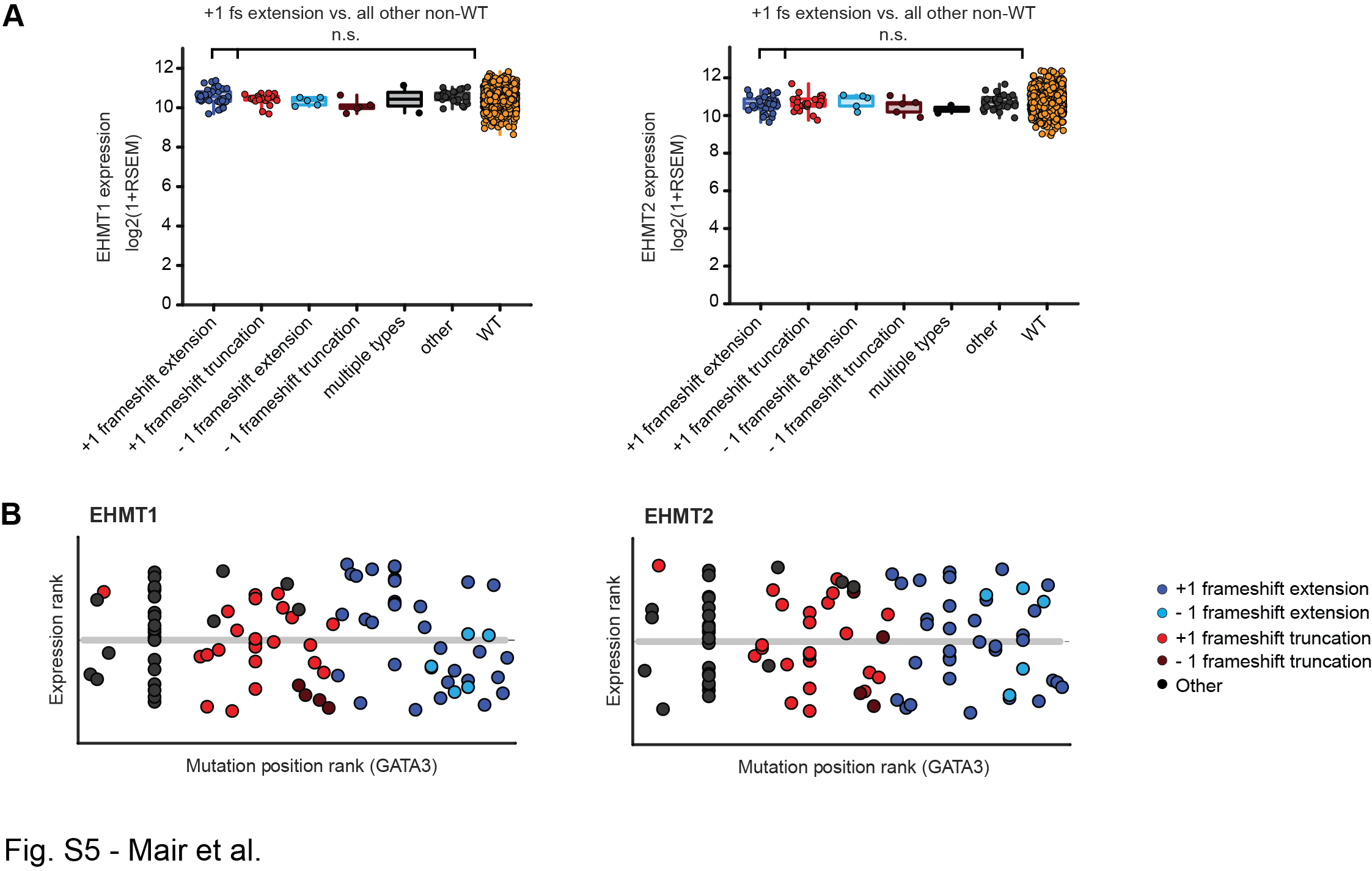

Supplement: S5 Fig — (A) Association between GATA3 mutations and EHMT1/2 gene expression in patient data. Expression values are the normalised RSEM values provided by TCGA. n.s., not significant by Wilcoxon test. (B) Association between mutation position and expression of EHMT1 and EHMT2. Horizontal axis shows ranked position of mutations along the GATA3 gene. On the vertical axis, ranked normalised expression values are displayed. These values are then segmented as described. Mutations are coloured according to category. (TIF) [file pgen.1006279.s005.tif]

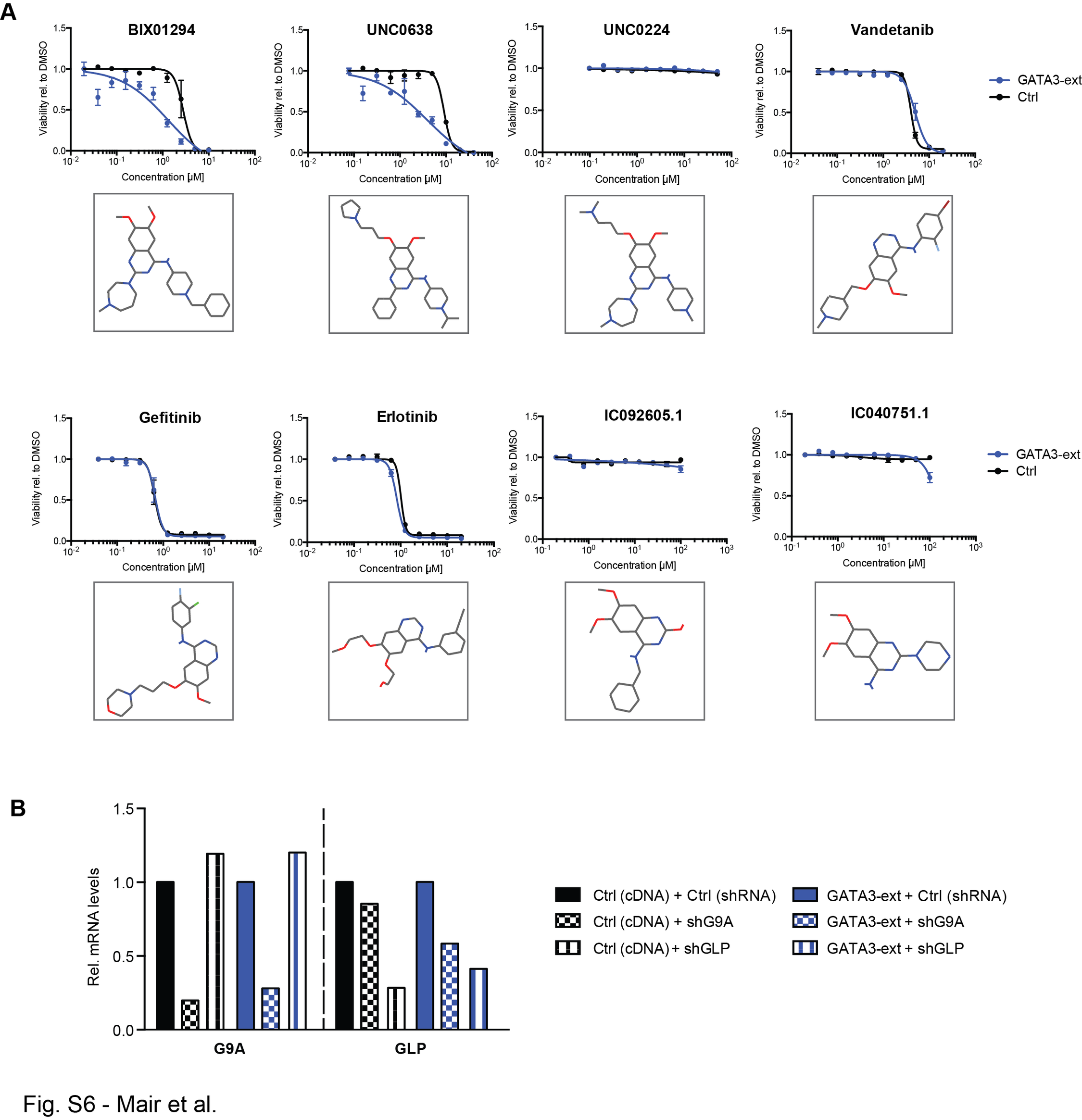

Supplement: S6 Fig — (A) DRCs accompanying Fig 4G. MCF10A control and cells expressing GATA3-ext were treated with the indicated concentrations of structurally related quinazoline compounds for 4 days. Cell viability was measured and normalised to a DMSO control. The graphs show the mean of triplicate measurements. Error bars indicate SEM. Boxes show compound structures. (B) G9A and GLP mRNA levels in MCF10A cells transduced with shRNAs were analysed by qRT-PCR. Values were normalised to GAPDH and displayed relative to parental cells (i.e. Ctrl (cDNA) or GATA3-ext) transduced with shRNA control. (TIF) [file pgen.1006279.s006.tif]

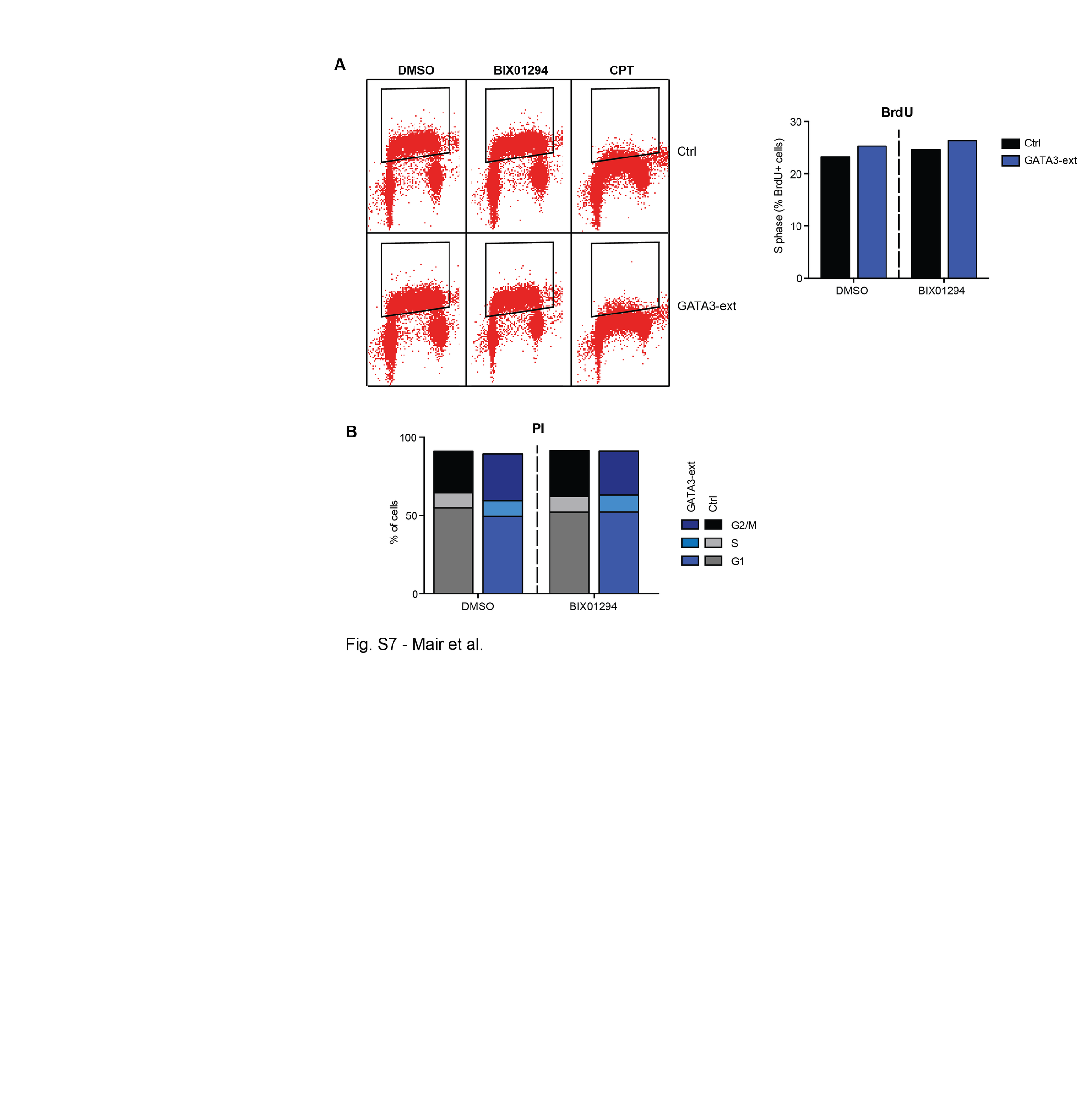

Supplement: S7 Fig — (A) Cells were treated with DMSO, BIX01294 (1μM) for 3 days or Camptothecin (CPT, 1μM, positive control) for 4 hours. Bromodeoxyuridine (BrdU) was added, cells were fixed, stained with an antibody recognising BrdU and analysed by FACS. (B) Cells were fixed, stained with propidium iodide (PI) and analysed by FACS. Cell cycle phases were derived from DNA content. (TIF) [file pgen.1006279.s007.tif]
